# Assessing the availability, readability, and content of online patient Education materials for cancer pain interventions: A cross-sectional analysis of major cancer center websites

**DOI:** 10.1016/j.inpm.2025.100633

**Published:** 2025-08-21

**Authors:** Meha Aggarwal, Marshall Yuan, David Hao

**Affiliations:** a Robert Wood Johnson Medical School, Piscataway, NJ, 08854 United States; b Department of Anesthesia, Critical Care and Pain Medicine, Massachusetts General Hospital, Harvard Medical School, Boston, MA, 02115, United States

**Keywords:** Cancer pain, Patient education, Health literacy, Readability, Quality, Online health information

## Abstract

**Background:**

As cancer survival improves, chronic cancer-related pain is an increasing clinical concern. Interventional procedures offer targeted, opioid-sparing pain relief, yet the quality and readability of online educational materials about these options remain poorly understood.

**Objective:**

To evaluate the availability, quality, and readability of online educational resources on interventional cancer pain management available from National Cancer Institute (NCI)-designated cancer centers.

**Methods:**

We conducted a cross-sectional analysis of 65 NCI-designated clinical cancer center websites to identify patient-facing content discussing interventional cancer pain procedures. Eligible materials were evaluated for quality using the DISCERN instrument and for readability using seven validated metrics. Inter-rater reliability was assessed using the intraclass correlation coefficient (ICC). Statistical comparisons were performed using t-tests.

**Results:**

Only 20 of 65 cancer center websites (31%) contained relevant educational materials. Sixty qualifying texts were identified: 28 full articles and 32 substantial mentions (≥50 words). The mean DISCERN score was 37 ± 9, indicating poor quality. Articles scored significantly higher than substantial mentions (mean difference 9.4 points, p < 0.001). The ICC for DISCERN scores was 0.872 (p < 0.001), reflecting good inter-rater agreement. Readability analysis revealed an average reading level equivalent to the 11th grade across all metrics, significantly higher than the NIH-recommended 8th-grade level (p < 0.001). Substantial mentions were significantly more difficult to read than articles (p < 0.001).

**Conclusions:**

Online materials on cancer pain interventions are generally scarce, low in quality, and written above nationally recommended reading levels. These findings highlight the need for cancer centers to improve online education materials using plain language and health literacy tools to better support informed decision-making.

## 1 Introduction

Pain is a common and often debilitating symptom associated with cancer, affecting both patients undergoing active treatment and those who have entered remission. Studies have shown that chronic pain related to cancer can persist for years after treatment has ended, significantly impacting survivors' quality of life and daily functioning [[1], [2], [3], [4]]. This pain can result from factors such as tumor progression, treatment-related nerve damage, or surgery, and may affect individuals with widespread disease as well as long-term survivors who are otherwise cancer-free. With advancements in early detection, targeted therapies, and supportive care, cancer survival rates have markedly improved [5,6]. As a result, an increasing number of patients may face chronic pain, whether during treatment or in survivorship, and require sustained management strategies. While opioids have traditionally been a cornerstone of cancer pain management, their long-term use raises concerns regarding safety, dependence, and diminishing effectiveness. Consequently, interventional approaches, such as nerve blocks, spinal drug delivery systems, and neurolytic techniques, are playing an increasingly prominent role [[7], [8], [9]]. These minimally invasive procedures can help control pain, particularly when conventional therapies are ineffective or limited by adverse effects.

For patients to make informed decisions about procedures, comprehensive and accessible education is essential. Many patients rely on the internet as a primary source of health information, but the quality of these online resources varies widely. Issues such as poor readability, incomplete content, and questionable accuracy can hinder patients' understanding and compromise shared decision-making [10,11]. Although the NIH recommends that educational materials be written at a 7th to 8th-grade reading level, most procedural content remains complex and filled with medical jargon, limiting patients’ ability to comprehend and trust the information available online [12].

Through this cross-sectional study, we aim to systematically evaluate the readability, quality, and comprehensiveness of online educational materials available on major cancer center websites regarding interventional approaches to cancer pain. Our goal is to identify gaps and opportunities to enhance patient education, thereby supporting more informed and empowered healthcare decisions.

## 2 Methods

### 2.1 Website selection

Websites of National Cancer Institute (NCI)-designated cancer centers were selected as the primary sources of patient-facing educational content. Cancer centers earn NCI designation by meeting several criteria, which include strong basic laboratory research, a track record of innovative clinical research, and a commitment to community outreach. Additionally, NCI-designated cancer centers appear to have stronger survival and mortality outcomes than non-designated cancer centers [[13], [14], [15]].

Of the 72 NCI-designated cancer centers, 8 are Clinical Cancer Centers and 57 are Comprehensive Cancer Centers, both of which provide clinical care. The remaining 7 Basic Laboratory Cancer Centers focus solely on preclinical research and were excluded.

### 2.2 Data collection

Websites of the 65 eligible cancer centers were manually reviewed for patient-oriented educational content related to interventional cancer pain management. We focused on a predefined set of procedures selected for their established or emerging use in the treatment of cancer-related pain, as supported by published guidelines and clinical practice [16,17]. These included both neuraxial and peripherally targeted interventions, as well as ablative and neuromodulatory techniques. Searches used keywords such as “celiac plexus block,” “superior hypogastric block,” “ganglion impar block,” “intercostal nerve block,” “cryoablation,” “chemical neurolysis,” “cordotomy,” “intrathecal drug delivery,” “spinal cord stimulation,” “dorsal root ganglion stimulation,” “peripheral nerve stimulation,” “vertebroplasty,” “kyphoplasty,” “cementoplasty,” “myelotomy,” and “dorsal root entry zone lesioning.” Patient education sections, dropdown menus, and subpages were explored for relevant content. Materials were included only if they provided explanatory content rather than brief or passing mentions.

### 2.3 Inclusion criteria

Materials were included if they met either of the following: (a) *dedicated article* discussing a specific intervention, including procedural details, indications, risks, and benefits; or (b) *substantial mention* of at least 50 words that provided more than a brief listing. The 50-word threshold was a subjective cutoff used to help categorize content with meaningful explanatory value. Materials were excluded if they contained *minimal mentions* (fewer than 50 words without meaningful context), were *irrelevant* (e.g., general overviews of cancer pain without intervention-specific information), or if the same content appeared redundantly across multiple pages of the website.

### 2.4 Quality analysis

Patient-facing materials were assessed using the DISCERN instrument, a validated tool for evaluating the reliability and comprehensiveness of consumer health information [18]. Two reviewers, MA and MY, independently scored each text using the 16-item DISCERN questionnaire, which includes 15 items measuring content quality and one item assessing overall quality. Each item was scored on a scale from 1 to 5, with 1 = “no,” 5 = “yes,” and 3 = “partially.” Final scores were calculated as the average of both reviewers’ assessments. The third author, an interventional pain physician and director of a cancer pain program, was available to address any questions. They also reviewed the scoring framework and results to ensure accuracy and appropriate application to interventional pain content, and monitored for outlier scores.

### 2.5 Readability analysis

Readability was evaluated using seven validated readability metrics available through the online tool readable.com.

Flesch-Kincaid Grade Level (FKGL) estimates the U.S. school grade level based on sentence length and word complexity.

Flesch Reading Ease (FRE) scores range from 1 to 100 based on average sentence length and word complexity, with higher scores indicating easier-to-read text.

Gunning Fog Index (GFI) assigns a grade level based on sentence complexity and polysyllabic word use, with scores above 17 suggesting graduate-level text.

The Simple Measure of Gobbledygook (SMOG) Index estimates the years of education needed to comprehend the material, based on the polysyllabic word count.

Automated Readability Index (ARI) evaluates word and sentence length, with higher scores corresponding to higher grade-level requirements.

Coleman-Liau Index similarly uses characters per word and words per sentence to estimate readability.

Fry Readability Formula (FRF) calculates grade level using sentence length and syllable count and correlates closely with other such metrics, including the Flesch Reading Ease.

### 2.6 Statistical analysis

All continuous variables were calculated as mean ± standard deviation. Inter-rater reliability of DISCERN scores was assessed using the intraclass correlation coefficient based on a two-way mixed effects model with absolute agreement. A two-sample *t*-test was used to identify differences in metric means between articles and substantial mentions. A one-sample *t*-test was used to identify the difference between the estimated readability of the included texts and the NIH-recommended 8th-grade reading level. Significance was defined as α < 0.05. All statistical analyses were performed using IBM SPSS 30 Statistics Premium Edition.

## 3 Results

### 3.1 Website selection

**Fig. 1:**
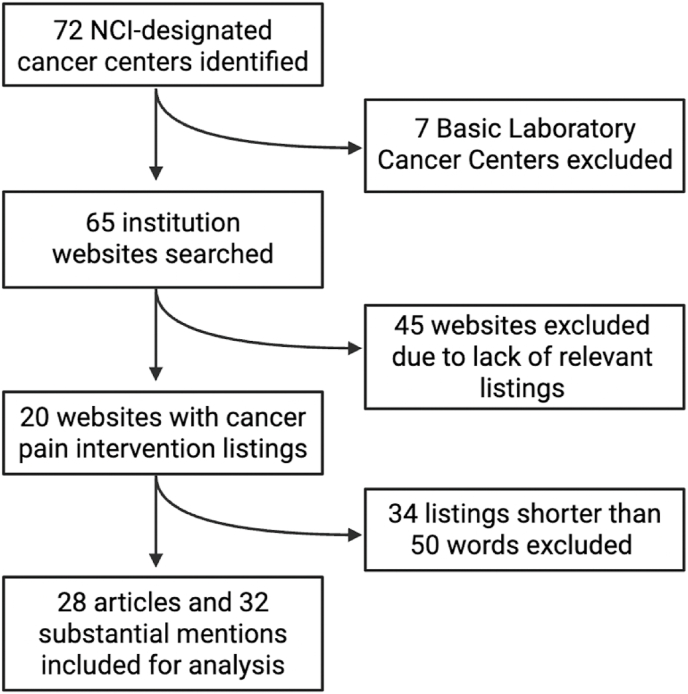
Summary of website selection process.

Of the 65 cancer center websites reviewed, only 20 contained any content specific to cancer pain interventions. These 20 websites were then examined in greater detail for articles or mentions related to such interventions. A total of 94 mentions were identified, of which 34 were excluded for being under 50 words in length. The remaining 28 articles and 32 substantial mentions were included in the final analysis and evaluated using the aforementioned quality and readability metrics. Fig. 1 outlines the process of identifying eligible materials.

### 3.2 Quality analysis

The DISCERN tool was used to evaluate all 28 included articles and 32 substantial mentions. Both reviewers’ scores were then assessed for inter-rater reliability. Intraclass correlation coefficient for overall scores was 0.872 (*p* < 0.001), indicating good agreement (0.75 to < 0.9) between raters [19]. The overall mean DISCERN score was 37 ± 9 across all texts, ranging from 24.5 to 61. Literature suggests that a score of 37 is considered “poor,” with scores below 27 being “very poor.” The maximum DISCERN score in this study is 61, which is considered “good.” Neither author rated any text as “excellent,” generally defined as scores over 63 [[20], [21], [22], [23]].

**Fig. 2:**
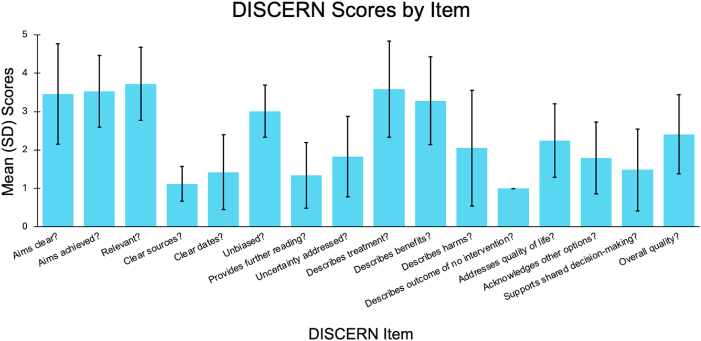
DISCERN Scores by Item. Bars represent mean scores. Error bars represent ±1 standard deviation. Item 12, “Describes outcome of no intervention?”, received a score of “1” across all responses and thus does not include error bars.

We then analyzed both reviewers' responses to each item. The DISCERN instrument has 16 questions; the first section (questions 1–8) assesses reliability, the second section (questions 9–15) assesses quality, and question 16 assesses overall rating. Fig. 2 displays the mean ± SD response to each question, highlighting the distribution of the reviewers’ responses across all articles and substantial mentions.

Finally, articles and substantial mentions were analyzed as separate groups. On average, articles performed 9.4 points higher than substantial mentions using the DISCERN tool (*p* < 0.001).

### 3.3 Readability analysis

Included texts were assessed for readability using the previously described metrics. Articles and substantial mentions were first analyzed as a combined group. The mean FRE was 48 ± 16, indicating a “difficult” or college-level readability [24]. Both the FKGL and ARI averaged 11 ± 3, indicating that the materials were written at approximately an 11th-grade reading level. These findings were corroborated by a mean GFI of 14 ± 3, a Coleman-Liau index of 12 ± 2, and a FRF score of 10 ± 3. The mean SMOG Index was 13 ± 2, further suggesting that the average readability exceeded the level of a high school graduate.

Using the mean values from FKGL, ARI, GFI, Coleman-Liau, FRF, and SMOG Index, the included cancer pain education materials were found to be significantly more difficult to read than the NIH-recommended 8th-grade reading level (*p* < 0.001 for all comparisons).

**Fig. 3:**
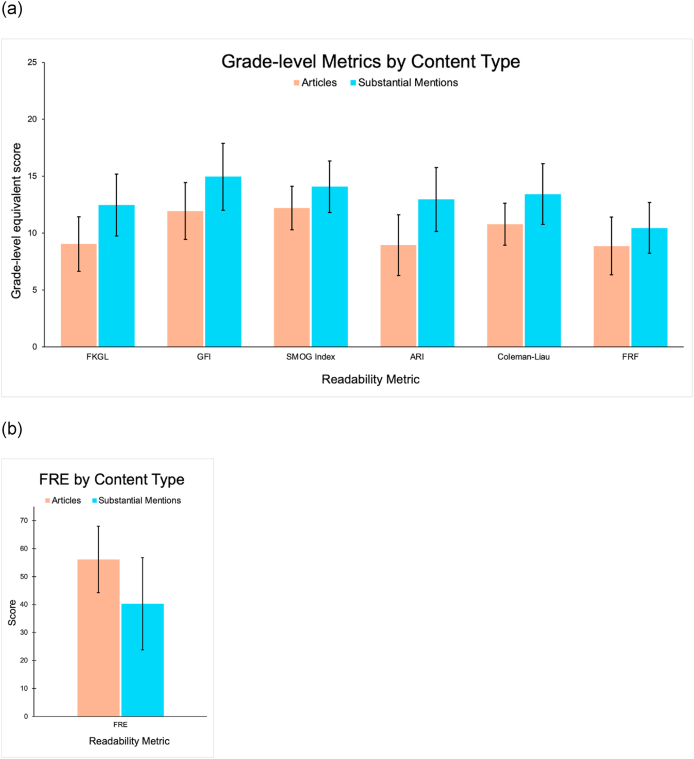
(a) Grade-level Metrics by Content Type. (b) FRE by Content Type. Bars represent mean scores. Error bars represent ±1 standard deviation.

Articles and substantial mentions were then analyzed separately, with means ± SD for each group shown in Fig. 3. Across all readability metrics, articles were significantly easier to read than substantial mentions (FRF *p* = 0.041; *p* < 0.001 for all other metrics). When comparing articles alone to the NIH-recommended 8th-grade reading level, four of six grade-level indicators were significantly above this threshold (FKGL *p* = 0.029; GFI *p* < 0.001; SMOG Index *p* < 0.001; Coleman-Liau *p* < 0.001). ARI (*p* = 0.073) and FRF (*p* = 0.113) did not show a statistically significant difference. Compared to the same standard, substantial mentions alone had significantly higher reading level than the NIH recommendation across all six metrics (*p* < 0.001 for all).

## 4 Discussion

In this study, we evaluated the availability, readability, and quality of online patient educational materials related to interventional cancer pain management using a series of validated tools and scores. Our analysis revealed a paucity of cancer pain intervention information across the assessed cancer websites, with only 20 of 65 NCI-designated clinical cancer centers providing relevant content.

Moreover, the materials that were available demonstrated generally poor quality, with a mean DISCERN score of 37. All resources also exhibited poor readability, requiring reading levels substantially above the NIH-recommended 8th-grade standard, which could hinder patient comprehension.

These findings raise serious concerns about the quality of publicly accessible cancer pain education. With an average DISCERN score of 37 ± 9, classified as poor, none of the materials reached the excellent quality threshold, and several were rated very poor with scores below 27. This suggests that many online health resources may not meet the standards necessary to support informed decision-making. Moreover, NCI-designated cancer centers meet strict standards for their classification and usually have superior survival and mortality outcomes; it is thus plausible that the websites included in this article are of higher quality than the average online patient literature. Given patients’ growing reliance on the internet for health information, poor quality content risks misinformation and misunderstanding about cancer pain interventions, which could adversely affect clinical outcomes.

Certain DISCERN criteria were consistently problematic. Items four, clarity of information sources, and five, date of information, score among the lowest, indicating frequent omission of citations and publication dates, which reduces transparency and trustworthiness. Item seven, availability of further reading, also scored poorly, limiting the patient's ability to seek out additional information. Similarly, items twelve, description of consequences of no intervention, and fifteen, promotion of shared decision making, received low scores. This underscores the tendency to present interventions as potentially favorable without acknowledging alternatives or encouraging patient involvement.

When comparing articles to substantial mentions, articles scored on average 9.4 points higher on the DISCERN scale. This likely reflects differences in content depth, editorial oversight, and expert involvement. Full articles are more likely to be structured, reviewed, or possibly authorized by subject experts, whereas substantial mentions often lack similar detail or scrutiny. This discrepancy highlights that not all online written materials are of equal quality; patients who rely on substantial mentions for information regarding cancer pain interventions may be particularly vulnerable to misinformation or an imbalanced presentation of risks and benefits. Notably, we opted to include mentions that were at least 50 words long as a subjective cutoff, aiming to analyze only meaningful content. Patients who rely on even shorter information bytes may be consuming poorer quality information than our findings suggest.

Readability across materials was poor, with an average reading level of 11th grade. With a mean FRE of 48 ± 16, the included texts fall into the “difficult” category, suggesting that a substantial portion of the population may struggle to comprehend the information. Grade-level metrics corroborate this finding: the average reading level across all tools—FKGL, ARI, GFI, Coleman-Liau, FRF, and SMOG—ranged from 10th-to 14th-grade, well above the 8th-grade level recommended by the NIH for patient education materials. All comparisons to this standard were statistically significant (*p* < 0.001), further emphasizing the discrepancy. When articles were analyzed alone, only four out of six metrics demonstrated significantly higher reading levels compared to the NIH recommendation. By contrast, all six metrics indicated that substantial mentions were significantly harder to read when compared to the 8th-grade reading level. This discrepancy suggests that dedicated articles might be ironically easier to comprehend for patients than short paragraphs, which may include medical jargon and complex, condensed information.

These elevated readability levels are particularly problematic in the context of health literacy. Research shows that nearly nine out of ten adults in the United States struggle with health literacy when faced with complex medical information [25]. Educational materials that cater to college-educated patients risk alienating a large segment of the population, potentially leading to reduced adherence to treatment recommendations and increased confusion. By failing to accommodate patients at high risk for poor health literacy. such as individuals from lower socioeconomic backgrounds, ethnic minorities, and older patients, we risk exacerbating health disparities. When patients cannot understand the available options for cancer pain relief, they are potentially less likely to participate in shared decision-making or advocate for themselves.

Our study builds on prior research showing limitations in online health content by focusing specifically on cancer pain intervention content, an area that remains largely unexplored. Existing literature has documented similar deficits in other fields: patient education materials on ophthalmologic surgery, interventional radiology, and pediatric procedures, among others, are frequently written at reading levels well above the NIH's recommended 8th-grade reading level. This reflects a broader disconnect between recommended readability standards and the actual complexity of online health information [[26], [27], [28]].

These findings reinforce the urgent need for high-quality, accessible educational materials addressing cancer pain interventions. Given the complexity of interventional pain management, patient comprehension is foundational to informed consent and optimal clinical outcomes. When educational resources lack readability and reliability, patients are at increased risk of misunderstanding critical information or foregoing beneficial interventions. As digital platforms become a primary source of health information, ensuring that online content is understandable, accurate, and reliable is not only a practical priority but also an ethical imperative. Without accessible, high-quality information, these procedures may remain out of reach for the most vulnerable populations.

This study has several limitations that should be considered when interpreting results. One notable consideration is that texts were scored by non-physician authors. Both independent reviewers were medical students who received training in applying the DISCERN instrument in the clinical context of celiac plexus block. The third author, an interventional pain physician and director of a cancer pain program, did not independently score the materials but was available to address questions and reviewed the scoring framework and results for accuracy, appropriate application to clinical content, and outlier detection. Although the reviewers did not have the same depth of clinical experience, most DISCERN items assess general principles of reliability, sourcing, and completeness of information, making it unlikely that this limitation materially affected the findings.

There are further limitations in this study. Our analysis was restricted to the websites of National Cancer Institute-designated centers, which may limit the generalizability of our findings to other types of cancer care providers. Additionally, we applied a subjective threshold of 50 words to define “substantial mentions”, which may have excluded potentially relevant content. The readability assessment relied on text-based metrics and did not account for visual elements, multimedia features, or interactivity, all of which can potentially influence patient comprehension and engagement. Similarly, while the DISCERN instrument is validated for evaluating written health information, it may not capture the specific nuances of interventional cancer pain procedures, such as procedural risks or technical details. Finally, this cross-sectional study focused on content availability, quality, and readability at a single point in time.

## 5 Conclusions

This study highlights significant gaps in the availability, quality, and readability of online educational materials related to interventional cancer pain management. The majority of existing content is limited in scope, low in quality, and written at a reading level far above national recommendations, posing a barrier to informed patient participation. Further research is required to understand how educational content influences patient decision-making and uptake of interventional procedures. Future studies should also evaluate multimedia formats, which were beyond the scope of this analysis, but increasingly shape how patients engage with health information.

To support comprehensive, patient-centered cancer pain care, coordinated efforts are essential. Cancer centers should prioritize reviewing and revising existing educational materials to ensure accuracy, completeness, and accessibility. Moving forward, cancer centers should adopt plain language principles and validated health literacy tools to ensure that all patients, regardless of background, can understand and act on the information provided.

## Funding

There are no funders to report for this submission.

## Declaration of competing interest

The authors declare that they have no known competing financial interests or personal relationships that could have appeared to influence the work reported in this paper.
